# Pediatric Presentations of Granulomatosis With Polyangiitis: A Double Case Study

**DOI:** 10.1155/crdm/6052518

**Published:** 2025-03-14

**Authors:** Marina Handal, Amit Sharma, Mara Ernst, Krystina Khalil, Eduardo Weiss

**Affiliations:** ^1^Department of Dermatology, Nova Southeastern University KP-COM, Fort Lauderdale, Florida, USA; ^2^Department of Dermatology, Larkin Community Hospital Palm Springs Campus, Hialeah, Florida, USA; ^3^Department of Dermatology, Hollywood Dermatology & Cosmetic Specialists, Hollywood, Florida, USA

## Abstract

Granulomatosis with polyangiitis (GPA) is an ANCA-associated necrotizing vasculitis that causes granulomatous inflammation of small vessels in the respiratory tract and mucosa; GPA in childhood is a rare occurrence that presents distinctly as either a chronic, granulomatous disease that is clinically localized or as an acute vasculitis with rapidly progressive pulmonary or renal hemorrhage. We present two distinct cases of GPA in pediatric patients whose diagnoses were confirmed according to clinical presentation and scoring guidelines offered by the ACR/EULAR GPA Criteria. Despite a negative cANCA result, Patient 1 demonstrated a score of 9 based on the ACR/EULAR criteria for GPA diagnosis. This was based on the patient's physical examination, which revealed tender nodules and plaques along the face as well as a crusted ulceration in the left concha. A punch biopsy of the left lateral forehead revealed necrotizing angiitis with neutrophil-predominant inflammatory infiltrate and giant cells on pathological analysis. In contrast, Patient 2 displayed a score of 13 as reflected in the extent of systemic disease involvement, with ulcerations and nodules scattered along the torso, extremities, and genitalia. Laboratory workup revealed ANCA positivity. Additionally, this patient experienced granuloma formation of the right optic nerve, ethmoid sinus infiltration with damage to the nasal septum, and bilateral cavitary masses on CXR. There is a paucity of data in characterizing GPA in childhood, as evidence is based on small cohort studies and case reports in this unique demographic. The clinical presentations in our report underscore the need for early disease detection and comprehensive workup, as timely diagnosis and optimal treatment regimens may improve the prognoses of pediatric patients with GPA.

## 1. Introduction

Granulomatosis with polyangiitis (GPA) is a pauci-immune systemic vasculitis characterized by necrotizing inflammation of small to medium sized vessels, generally seen in adults ages 40–60 years old. Pediatric onset of GPA is rare, as the incidence rate of disease in children ≤ 18 years is 1.8; 1,000,000 [1]. The classic pathologic triad of GPA remains applicable to pediatric patients, with disease manifestations afflicting the nasopharynx, lungs, and kidneys [1]. Patients often present with nasal septum perforation and sinusitis resulting in saddle-nose deformity. Furthermore, diffuse alveolar hemorrhage and glomerulonephritis can be seen resulting in increased morbidity and mortality. Etiologically, the granulomatous inflammation pathognomonic of GPA may result from a T-cell mediated hypersensitivity reaction [2]. Specifically, cytoplasmic antineutrophil autoantibodies (c-ANCA) against major antigen proteinase 3 (PR-3) is a specific and sensitive marker for this disease and can be detected in up to 90% of systemic disease cases [3]. GPA is treated with a combination of high dose glucocorticoids and oral and intravenous cyclophosphamide for 3–6 months. Rituximab, a monoclonal antibody, may also be helpful in preventing disease relapse by targeting CD20 antigen on B cells without clearing plasma cells from circulation [2]. Compared to GPA onset in adults, pediatric patients experience clinical symptoms across differing organ systems and at varying frequencies [4]. We present two unique cases describing rare and clinically complex, multisystem GPA in pediatric patients.

## 2. Case Presentations

### 2.1. Patient 1

**Figure 1:**
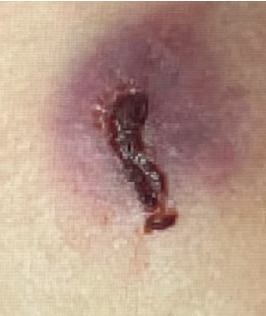
Tender, ecchymotic nodule with central ulceration and overlying hemorrhagic crust of the right zygoma.

**Figure 2:**
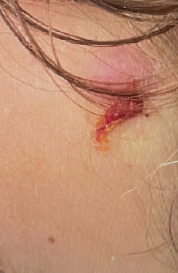
Ill defined, superficial eroded plaque with crust.

**Figure 3:**
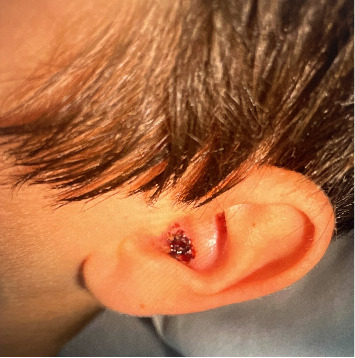
Ulcerated plaque with overlying hemorrhagic crust in the left conchal bowl.

**Figure 4:**
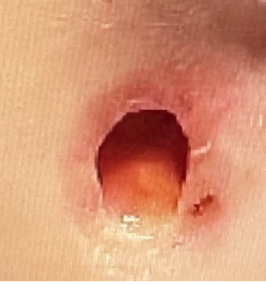
Progression of ecchymotic nodule into ulceration with sharply marginated borders.

A 13-year-old male presented to the emergency department (ED) with tender, eroded nodules on the face that enlarged over one month. During that time, the patient was also experiencing concomitant fevers, sinusitis, dysuria, and hematuria. He was previously treated for sinusitis and balanitis with amoxicillin, topical steroids and mupirocin ointment without improvement. Physical examination revealed an edematous, eroded, purpuric plaque with central hemorrhagic crust over the right zygoma, an eroded plaque over the left lateral forehead, and ulceration with overlying crust in the left concha (Figures 1, 2, and 3). Six days following admission, the lesion on the right zygoma progressed to an ulceration (Figure 4).

**Figure 5:**
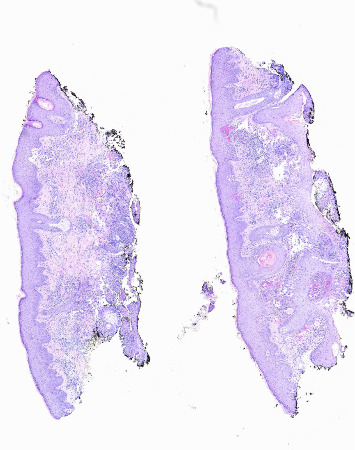
Punch biopsy of left lateral forehead. Low power revealing inflammation in the dermis with a vascular infiltrate.

**Figure 6:**
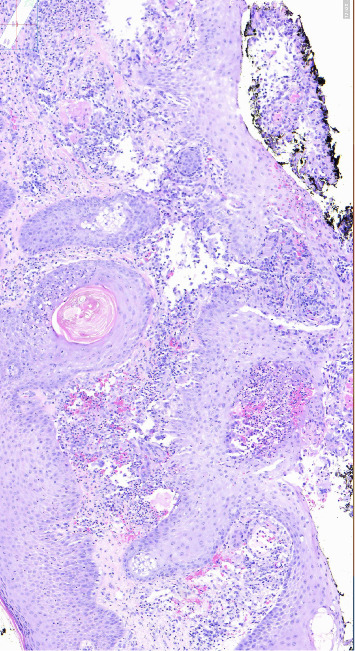
High power revealing a necrotizing angiitis with neutrophil predominant infiltrate in small to medium sized vessels.

**Figure 7:**
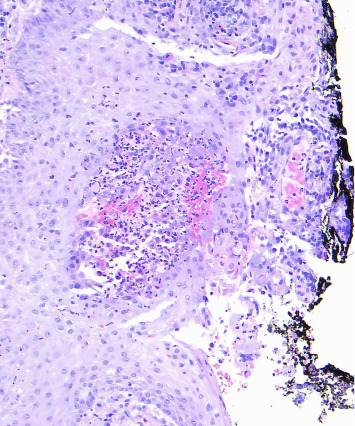
High power revealing red cell extravasation and extravascular granulomas with scattered giant cells.

Laboratory workup revealed CRP of 8.22 mg/dL, ESR of 42 mm/hr, and ANC of 14.70 ∗ 10^3^. ANCA test specifically serine protease-3 autoantibody (ANTI-PR3) was negative. Urinalysis revealed pyuria and hematuria, and respiratory panel was positive for rhinovirus. CT of the sinuses demonstrated subcutaneous nodular and diffuse pansinusitis. RUQ-ultrasound found evidence of bladder outlet obstruction and debris within the urinary bladder. CT chest revealed cavitary pulmonary nodules up to 2.4 cm. Punch biopsy from the left lateral forehead displayed necrotizing angiitis with neutrophil-predominant inflammatory infiltrate in small and medium-sized dermal vessels, red cell extravasation, and extravascular granulomas with scattered giant cells (Figures 5, 6, and 7). Despite the negative cANCA result, the combination of the pathology and clinical findings supported a diagnosis of GPA. During admission, the patient was treated with intravenous steroids and rituximab, then switched to oral prednisone 30 mg twice daily with appropriate taper upon discharge. Nine months later, he remains on prednisone 2.5 mg every other day and mycophenolate mofetil.

### 2.2. Patient 2

**Figure 8:**
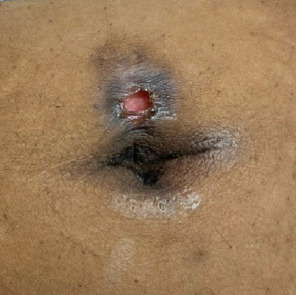
Clean ulceration with surrounding areas of hyperpigmentation and lichenification.

**Figure 9:**
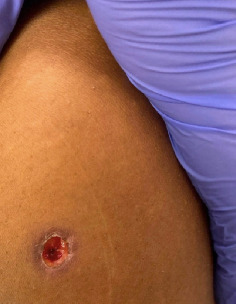
Ulceration with thin surrounding area of hyperpigmentation.

A 16-year-old female presented to the ED with nodules and ulcerations on the torso, upper extremities, and genitalia. Additionally, she reported fever, ongoing epistaxis, productive cough, dyspnea with exertion, facial pain, and unintentional weight loss. She had previously been diagnosed with acute parotitis treated with clindamycin and steroids, but returned to the ED for ongoing symptoms. On physical examination, the patient was febrile, tachycardic, and hypertensive. An ulcerated plaque with surrounding hyperpigmentation was located immediately above the umbilicus; a deep ulceration at the left shoulder and multiple nodules along the right upper extremity were also noted (Figures 8 and 9).

Laboratory workup revealed ANCA positivity with elevated ANC 10.14, ESR 145 mm/hr, and CRP 16.58 mg/dL. An ophthalmologic exam revealed decreased function of the right optic nerve with granuloma formation without evidence of uveitis or vasculitis. CT scan of the sinuses revealed osseous dehiscence of the ethmoid sinus, destructive loss of the nasal septum, and suspicions of subperiosteal abscess or granuloma. Bilateral cavitary masses were seen on CXR and bronchoscopy displayed bronchiolar hemorrhage. Renal ultrasound subsequently demonstrated kidney enlargement without hydronephrosis. Punch biopsies taken from the left elbow and shoulder displayed similar findings as in patient one. Treatment consisted of pulse steroids for 3 days followed by prednisone 30 mg twice daily for 2 weeks, a 4-week course of rituximab, and a 2-week course of antibiotics. Six months later, the patient's renal function returned to normal, although she continues to have hearing loss and is maintained in remission on prednisone 10 mg and mycophenolate mofetil with plans to begin using hearing aids in the near future.

## 3. Discussion

Childhood-onset GPA is a pauci-immune necrotizing vasculitis that can present with differing symptomatology than those seen in adult cases. The 2022 American College of Rheumatology/European Alliance of Association For Rheumatology (ACR/EULAR) GPA criteria offers a sensitive and specific framework in classifying small and medium-vessel vasculitis. This criterion defines pediatric GPA as a diagnosis in patients under the age of 18 and displaying clinical and laboratory signs of disease. The three primary clinical criteria are nasal involvement, conductive or sensorineural hearing loss, and cartilaginous deformities of the nose, ears, or endobronchial system. The criteria also entail laboratory and biopsy findings, including ANCA positivity, histopathologic evidence of granulomatous inflammation, and pulmonary nodules or cavitations on chest imaging. Mastoiditis, pauci-immune glomerulonephritis and blood eosinophil count ≥ 1 × 10^9^/L are also considered. Although ANCA positivity is a major identifier of disease, GPA patients may lack the marker so long as they share the additional features of disease [4].

Clinically, GPA afflicts multiple organ systems in pediatric patients, ranging from malaise and weight loss, to mucocutaneous ulcers or nodules and signs of renal failure [5]. It is important to recognize that signs of renal failure include hypertension, proteinuria, hematuria, and elevated creatinine [5]. Additionally, ENT manifestations may be the most frequently appearing in addition to constitutional symptoms [6]. Children also experience higher rates of hospitalizations and hematologic complications like leukopenia, neutropenia, and hypoglobulinemia [1].

A meta-analysis conducted to assess the clinical findings and outcomes of patients with childhood-onset GPA revealed that > 90% of pediatric patients were ANCA positive and most frequently displayed ENT, renal, and respiratory tract symptoms [7]. Additionally, manifestations such as alveolar hemorrhage and lung nodules are especially prominent in childhood GPA cases, with a prevalence over 60% [8]. Moreover, saddle nose-deformity and subglottic stenosis are complications specific to GPA in pediatric patients, with approximately 10% of patients under the age of 18 developing these findings [7]. In regards to correlation of disease with geographic origin, 70% of childhood-onset GPA occur in female patients, compared to adult GPA generally affecting males [7]. The median age of diagnosis is 14 years old and 55% of cutaneous manifestations are more prevalent in European patients [7].

Applying the ACR/EULAR criteria to our report, both patients demonstrated renal manifestations along with aggressive sinus infiltration. However, clinicians must be cognizant of the ACR/EULAR criteria as cANCA positivity is not a requirement for disease classification in pediatric populations. In our patients, case 1 had a score of 9 and case 2 had a score of 13. A score of 5 or greater meets the criteria for GPA. Additionally, childhood GPA may further be distinguished by unique findings, such as subglottic stenosis or sinonasal disease [8]. Patient 1, demonstrated negative laboratory workup for cANCA. A repeat test was not completed due to the patient's significant findings that were consistent with the diagnosis of GPA. Patient 2 displayed more features of systemic involvement, including cavitary infiltrations, optic granulomas, and subperiosteal invasion. Additionally, this patient experienced a subsequent improvement in kidney function. This is critical as ESRD occurs in 12% of pediatric-onset GPA cases and is a more prevalent phenomenon among children overall as opposed to adult patients under 65 years old with GPA [1]. Both patients' confounding presentation underscores the need for tissue biopsy as a definitive means of GPA diagnosis, especially among the limited disease distribution in pediatric patients. The features of Patients 1 and 2 as well as their diagnostic challenges and treatment progression are compared alongside adult-onset GPA in Table 1.

Pediatric guidelines for GPA management have yet to be devised, thus posing as a limitation in the diagnostic and treatment processes for the cases detailed in this report. It is recommended that children with this diagnosis are treated according to the guidelines enforced for adult remission and maintenance. Consistent with the therapies administered in both of our cases, the treatment of GPA in pediatric patients involves recommendations by EULAR and the Canadian vasculitis research network (CanVasc) which consists of glucocorticoid treatment and cyclophosphamide or rituximab along with daily calcium and vitamin D supplementation; in cases of remission maintenance therapy, low-dose steroids coupled with azathioprine, rituximab, methotrexate, or mycophenolate mofetil are suggested for pediatric patients for a minimum of 24 months [11]. Prognostically, approximately 90% of patients with pediatric-onset GPA achieve remission; however, 61% of them are prone to clinical relapse. Additionally, children experience higher rates of hematologic complications in contrast to adults. Specifically, prognosis is affected by the onset of leukopenia, neutropenia, and hypogammaglobulinemia [1].

## 4. Conclusion

In managing childhood onset GPA, timely diagnosis and intervention are critical. Given the complexity and severity of this condition, it is important to diagnose and initiate treatment quickly. Healthcare providers should remain vigilant in recognizing signs and symptoms of GPA in children to ensure optimal therapeutic intervention and improve long term outcomes for a more favorable prognosis.

## Figures and Tables

**Table 1:** Clinical comparison of pediatric GPA vs. classic manifestations of adult-onset granulomatosis with polyangiitis.

	Clinical manifestations	Diagnostic findings	Treatment
Patient 1	-Tender eroded nodules on the face for 1 month -Fevers, sinusitis, dysuria, hematuria -Eroded plaque with hemorrhagic crust on right zygoma -Eroded plaque on left lateral forehead -Ulceration of the left concha	-CRP: 8.22 mg/dL -ESR: 42 mm/hr -ANTI-PR3: Negative -CT scan of the sinuses demonstrated subcutaneous nodular and diffuse pansinusitis -CT chest: pulmonary nodules -Biopsy: Neutrophil predominant necrotizing angiitis on pathology of left lateral forehead	-IV steroids + rituximab on admission -Switched to prednisone 30 mg PO + tapering on discharge -Prednisone 2.5 mg every other day maintenance + mycophenolate mofetil

Patient 2	-Ulcerations on torso, upper extremities, and genitalia -Ulcerated plaque with hyperpigmentation superior to umbilicus -Fever, productive cough, dyspnea with exertion, unintentional weight loss -Granuloma formation of the right optic nerve -Osseus dehiscence of ethmoid sinus + nasal septum -Bronchiolar hemorrhage with bilateral cavitary masses on CXR	-CRP: 16.58 mg/dL -ESR: 145 mm/hr -ANC: 10.14 -CT sinuses: osseous dehiscence of ethmoid sinus and nasal septum; subperiosteal abscess formation -Biopsy: Neutrophil predominant necrotizing angiitis on pathology of left elbow and shoulder	-Pulse steroids for 3 days -Transitioned to prednisone 30 mg/2x day -Initiated 4-week course of rituximab followed by 2-week antibiotic treatment for history of ongoing parotitis -Prednisone 10 mg maintenance + mycophenolate mofetil -Persistent hearing loss corrected with hearing aids

Adult GPA [9, 10]	-Classic GPA: Triad of upper airway, lung, and kidney damage + potential for any organ involvement -Renal involvement with mechanical ventilation in 2/3 of presentations -Nasal crust formation (75% cases) -Peripheral nervous system involvement (44% cases): weakness, sensory mononeuritis development, multifocal neuropathy -Orbital lesions most commonly pseudotumor or granulomatous inflammation + episcleritis/conjunctivitis	-Biopsy is the gold standard for diagnosis of GPA and is strongly recommended. -Diagnosis is based on a combination of serological tests and imaging studies. -Perform CBC, RFT, LFT, coagulation tests -Obtain ANCA, ESR, CRP results -CT preferred over conventional radiographs -MR imaging useful for cardiac and ocular investigation -Complete urinalysis with 24 h proteinuria collection -Anti-pentraxins (anti-PTX3) antibodies are a novel, upcoming diagnostic biomarker helpful in patients with negative serology for MPO- and PR3-ANCA	-Combined glucocorticoids and cyclophosphamide demonstrate success in patients attaining remission -Combination of methotrexate with glucorticoids also helpful -Rituximab and infliximab also utilized -Histopathologic subtypes of kidney involvement do not guide treatment decision -Kidney biopsy is useful in providing prognostic information

## Data Availability

The authors received no external input of data for this work.
